# Will Stopping Aβ Production Reverse the Damage in Alzheimer Disease?

**DOI:** 10.1371/journal.pmed.0020418

**Published:** 2005-11-15

Dementia is a common condition in the elderly; around 6% of people over 65 and up to 50% over 90 have some form of dementia, about half of which are due to Alzheimer disease (AD). The dementia caused by AD has an insidious onset and a progressive course with slow deterioration in cerebral function, initially affecting short-term memory and cognitive skills, and later speech, motor functions, and personality. Death usually occurs within four to eight years after diagnosis.

The aim of treatment is to reverse cognitive decline and improve behavioral and psychological functions. Key questions in Alzheimer research are how best to halt the progression of disease to maintain and if possible restore cognitive skills, and when to initiate such interventions in order to be effective.

AD is identified at autopsy by the presence of hallmark lesions in key regions of the brain. These lesions, known as amyloid plaques, are formed by the aggregation of small peptides, called amyloid β peptide (Aβ), that are produced when amyloid precursor protein (APP) is cleaved by the action of two enzymes, β-APP cleaving enzyme and γ-secretase. One approach to the treatment of Alzheimer is, therefore, limiting the production of Aβ from its precursor by inhibiting one or both of these enzymes. However, it is not yet clear whether this approach will prevent the brain lesions and cognitive symptoms from getting worse, and if it will then promote the removal of preexisting plaques and reverse cognitive decline.

To answer such questions, Joanna Jankowsky and colleagues have developed mice that produce Aβ at levels sufficient to induce severe amyloid burden by six months of age. The animals carry an additional transgene that acts as a switch to control when Aβ is produced. Commonly known as the tet-off system, the switch is turned off when the mice are fed tetracycline or its analog, doxycycline. Once given the drug, Aβ production in the brains of these mice diminishes by more than 95% of pretreatment levels within two weeks. This system, thus, mimics the effect of shutting down Aβ production with enzyme inhibitors that are being developed for use in human patients. [Fig pmed-0020418-g001]

**Figure pmed-0020418-g001:**
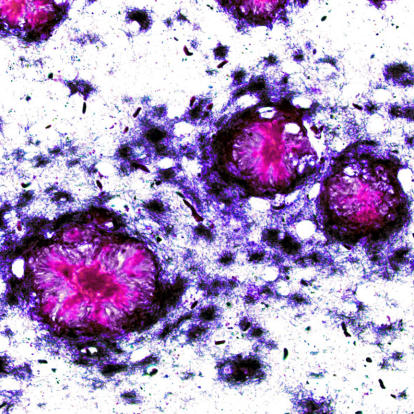
Amyloid plaques, shown here as false-color images, are highly stable structures in vivo

In the study, the researchers used doxycycline to switch off production of Aβ, and examined what happened to the amyloid pathology. Not surprisingly, the increase in number and size of amyloid lesions that normally occurs as the mice get older was completely prevented by suppressing Aβ production. However, the researchers also found no substantial clearance of preexisitng plaques, even after six months of treatment (one-quarter of the normal mouse lifespan).

What do these findings mean for human Alzheimer research? First, the study provides evidence that the lesions found in AD may be more difficult for the brain to repair than protein aggregates found in other diseases such as Huntington or prion disease. Second, the findings suggest that the removal of plaques, once formed, may require more than simply halting the production of the Aβ peptide. However, as with all animal models, there are differences in comparison to the human disease, leading to both over- and underestimation of the relative importance of an effect in humans. The researchers do not yet know whether the plaques formed in mice may be more resistant to clearance than those seen in human disease. Conversely, the human brain, unlike the murine one, may have a more efficient way of clearing amyloid plaques. What this study makes clear is that treatments directed at reducing Aβ peptide production in AD will likely be most effective when started as early as possible.

